# Phase I In Vitro Metabolic Profiling of the Synthetic Cannabinoid Receptor Agonists CUMYL-THPINACA and ADAMANTYL-THPINACA

**DOI:** 10.3390/metabo11080470

**Published:** 2021-07-21

**Authors:** Manuela Carla Monti, Eva Scheurer, Katja Mercer-Chalmers-Bender

**Affiliations:** Institute of Forensic Medicine, Department of Biomedical Engineering, University of Basel, 4056 Basel, Switzerland; manuela.monti@unibas.ch (M.C.M.); eva.scheurer@unibas.ch (E.S.)

**Keywords:** synthetic cannabinoid receptor agonists, in vitro metabolism, high resolution mass spectrometry, Compound Discoverer

## Abstract

Synthetic cannabinoid receptor agonists (SCRAs) remain popular drugs of abuse. As many SCRAs are known to be mostly metabolized, in vitro phase I metabolic profiling was conducted of the two indazole-3-carboxamide SCRAs: CUMYL-THPINACA and ADAMANTYL-THPINACA. Both compounds were incubated using pooled human liver microsomes. The sample clean-up consisted of solid phase extraction, followed by analysis using liquid chromatography coupled to a high resolution mass spectrometer. In silico-assisted metabolite identification and structure elucidation with the data-mining software Compound Discoverer was applied. Overall, 28 metabolites were detected for CUMYL-THPINACA and 13 metabolites for ADAMATYL-THPINACA. Various mono-, di-, and tri-hydroxylated metabolites were detected. For each SCRA, an abundant and characteristic di-hydroxylated metabolite was identified as a possible in vivo biomarker for screening methods. Metabolizing cytochrome P450 isoenzymes were investigated via incubation of relevant recombinant liver enzymes. The involvement of mainly CYP3A4 and CYP3A5 in the metabolism of both substances were noted, and for CUMYL-THPINACA the additional involvement (to a lesser extent) of CYP2C8, CYP2C9, and CYP2C19 was observed. The results suggest that ADAMANTYL-THPINACA might be more prone to metabolic drug−drug interactions than CUMYL-THPINACA, when co-administrated with strong CYP3A4 inhibitors.

## 1. Introduction

Synthetic cannabinoid receptor agonists (SCRAs) are a prominent class within the world of new psychoactive substances (NPS). In recent years, SCRAs, together with synthetic cathinones, were the predominantly seized classes of NPS in Europe [1]. SCRAs encompass a large variety of structurally diverse compounds with binding affinities to the cannabinoid receptors 1 and 2 (CB1 and CB2). Particularly via interaction with CB1, most SCRAs present cannabimimetic effects similar to Δ9-tetrahydrocannabinol (THC), the major psychoactive compound in cannabis [2,3,4]. The typically higher binding affinities of SCRAs as full agonists at CB1 and CB2, when compared to THC, are attributed to the often-observed increased potency, but also toxicity, of these compounds. Nevertheless, data on the pharmacology and toxicity of SCRAs is still limited [2,5,6]. Several cases of severe intoxication, including lethal outcomes, have been associated with the intake of SCRAs, thus underlining the public health threat posed by these compounds [7,8,9,10,11,12].

SCRAs are classified based on their chemical structure [2]. In recent years, many indazole- and indole-carboxamide-derived SCRAs have been reported, with 5F-MDMB-PINACA (5F-ADB), 5F-MDMB-PICA, and MDMB-4en-PINACA being frequently reported after detection in diverse formulations, ranging from shredded herbs that have been sprayed with SCRAs (“spice”), infused papers, e-liquids, and bulk powders [13,14,15]. Since the end of 2019, drug checking services in Switzerland have increasingly reported SCRAs fortified THC-low cannabis [16]. As these illicit products are generally sold as the nonaltered natural drug hemp, consumers unknowingly consuming SCRAs are clearly posed with an increased health threat. As the emergence of SCRAs on the drug market is constantly changing, as well as showing regional differences (for instance due to varying legal frameworks), it is important that analytical laboratories are constantly developing their analytical approach to SCRAs. Urine is a matrix that is often used for screening procedures in clinical and forensic toxicology due to favorable accessibility, higher concentrations of the substance of interest, and often longer detection windows when compared to blood. However, many SCRAs are known to be extensively metabolized, leading to a significant decrease or even lack of the parent compound in urine. As a consequence, metabolism studies identifying suitable target metabolites of NPS are inevitable [17,18,19,20].

CUMYL-THPINACA is classified as an indazole-3-carboxamide SCRA. A patent for CUMYL-THPINACA was issued in 2014 [21]. The cumyl-moiety is part of several SCRAs, as in, for example, CUMYL-BICA, 5F-CUMYL-PINACA, 5F-CUMYL-PICA, and CUMYL-4CN-BINACA [22]. The metabolism of several cumyl-bearing SCRAs has been investigated before [23,24,25,26], therefore the obtained results for CUMYL-THPINACA expand the current knowledge on the metabolism of members of this diverse subgroup. Considering its activity, Asada et al. synthesized CUMYL-THPINACA, finding strong activity at CB1 and CB2 [27]. This was confirmed via radioligand binding studies conducted by Schoeder et al. that showed high binding affinities of CUMYL-THPINACA at both CB1 (*K_i_* = 1.23 ± 0.20 nM) and CB2 (*K_i_* = 1.38 ± 0.86 nM) [28]. Even though these data on the affinity and activity of CUMYL-THPINACA exist, metabolic profiling, resulting in suggested biomarkers for the detection of the consumption of CUMYL-THPINACA, has, to the best of our knowledge, not been conducted yet.

ADAMANTYL-THPINACA, also referred to as ATHPINACA, is structurally related to CUMYL-THPINACA and AKB48 (APINACA). The adamantyl-moiety can be connected to the rest of the molecule, yielding two positional isomers of ADAMANTYL-THPINACA, which are referred to as isomer 1 [N-(1-adamantyl)] and isomer 2 [(N-(2-adamantyl)]. This study focusses on isomer 1, if not further specified. ADAMANTYL-THPINACA was first reported by EMCDDA’s Early Warning System after it appeared in Slovenia in 2015 [29], followed by Hungary in 2016 [30]. Recently, a study was published focusing on the metabolism of adamantyl-positional isomers of SCRAs, including first data on both isomers of ADAMANTYL-THPINACA. Metabolites were produced via incubation of pooled human liver microsomes (pHLM) and nine metabolites resulting from mono-, di-, and tri-hydroxylation were identified for isomer 1 of ADAMANTYL-THPINACA. Additionally, two glucuronidated metabolites were identified [31].

In this study, we present the phase I in vitro metabolic profiling of CUMYL-THPINACA and ADAMANTYL-THPINACA, applying two experimental set-ups. First, both SCRAs were incubated using pHLM, resulting in structural elucidation and identification of potential in vivo biomarkers of the detected metabolites. The incubation of active pharmaceutical ingredients with pHLM, amongst other in vitro models (such as human hepatocytes), is an established procedure for initial characterization of human metabolism [18,20,32] and therefore highly valuable for the study of SCRAs, for which information on the metabolism and suitable biomarkers is often lacking [20]. Metabolites as certified reference standards are often not available. Therefore, in vitro metabolism studies are a good alternative to incorporating metabolites into screening methods. Second, the cytochrome P450 enzymes (CYP) responsible for the phase I metabolism of the studied SCRAs were identified via incubation of a pallet of recombinant CYP isoforms (rCYP), thus expanding the present knowledge on the metabolism of ADAMANTYL-THPINACA as reported by Kadomura et al. [31]. Information on the metabolizing CYP isoforms gives the opportunity to predict the likelihood of metabolic drug−drug interactions or adverse events due to CYP polymorphisms [33,34,35,36,37]. A study conducted by Holm et al. showed that CYP3A4 was mainly responsible for the biotransformation of AKB48, an SCRA structurally related to ADAMANTYL-THPINACA. Nevertheless, specific CYP isoforms involved in the metabolism of SCRAs are often understudied and have, so far, not been investigated for CUMYL-THPINACA or ADAMANTYL-THPINACA.

Due to the diversity and large numbers of NPS emerging on the drug market, the rapid identification of target metabolites for screening procedures is urgently needed. High-resolution mass spectrometry (HR-MS) data analysis software is gaining importance, as in silico-assisted workflows enable higher throughput and are able to markedly facilitate metabolite identification [38,39]. In this study, data analysis was assisted by the Compound Discoverer (Thermo Fisher Scientific, Reinach, Switzerland) software, which has already been proven helpful for metabolite identification and structure elucidation in previously published studies [38,40,41,42,43].

## 2. Results and Discussion

### 2.1. Metabolite Identification for CUMYL-THPINACA and ADAMANTYL-THPINACA after Incubation with pHLM and rCYP

Functionality of the pHLM assay was assured by incubations of UR-144 (positive control) and subsequent detection of its N-(5-hydroxypentyl) and N-pentanoic acid metabolites. Negative controls did not result in any metabolite signals.

CUMYL-THPINACA and ADAMANTYL-THPINACA were extensively metabolized, resulting in a substantial decrease of the parent compound in the incubation mixture. Several metabolites resulting from mono-, di-, tri-hydroxylation, desaturation (most likely via hydroxylation followed by dehydration), and carbonylation, as well as combinations thereof, were identified. A summary of all detected metabolites and artefacts, and the results obtained via rCYP incubation, are shown in in Table 1 and Table 2 (for CUMYL-THPINACA) and Table 3 and Table 4 (for ADAMANTYL-THPINACA).

### 2.2. In-Source Water Loss of Metabolites

As a consequence of using electrospray ionization (ESI), in-source-fragmentation processes may occur [39,40,41,42]. For example, the observed alleged metabolites, presenting a mass shift of +13.9838 Da in comparison to the parent compound, may result from either hydroxylation in combination with desaturation (e.g., di-hydroxylation followed by dehydration) or carbonylation. However, the corresponding signals may also arise from in-source water loss, resulting from the cleavage of aliphatic hydroxyl-groups (e.g., at the 4-methyl-tetrahydropyran- and adamantyl-moiety). In-source water loss was considered as likely, where (i) a hydroxylated metabolite was detected, exhibiting a hydroxyl group at a position predestined for in-source water loss, (ii) a co-eluting signal was identified, presenting a dehydration-specific mass shift of −18.0153 Da (-H_2_O), and (iii) after fragmentation, when the type and position of biotransformation were identical for the hydroxylated metabolite and the alleged artefact. For example MC21, a metabolite produced by mono-hydroxylation at the 4-methyl-tetrahydropyran-moiety (i) was detected, but additionally a signal at the corresponding retention time (Rt) with mass shift of [M + H]^+^ −18.0153 Da was found (ii), which exhibits dehydration at the 4-methyl-tetrahydropyran-moiety (iii). Therefore, this signal was classified as an artefact (MCArt4).

The diversity in the hydroxylation patterns of metabolites, especially in cases of two or three concurrent hydroxylations, makes the evaluation of in-source processes highly complex. The observed results suggest that the susceptibility for in-source water loss considerably varies between aliphatic structures (e.g., adamantyl versus 4-methyl-tetrahydropyran). This becomes obvious when comparing the peak areas of genuine metabolites and the corresponding in-source artefacts. In the case of MA2 (hydroxylated at the adamantyl-moiety) the corresponding artefact (MAArt1) showed a 6.8 times higher signal than observed for MA2 itself. In comparison, MC21 (hydroxylated at the 4-methyl-tetrahydropyran-moiety) exhibited an in-source dehydration signal of roughly the same intensity as that observed for MC21. Additionally, positional isomers of hydroxylations within a moiety led to varying levels of observed water loss. For instance, when investigating the metabolite cluster MC8a–e (consisting of several co-eluting di-hydroxylated metabolites, bearing a hydroxyl-group at the 4-methyl-tetrahydropyran-moiety), in-source water loss varied from excessive (artefact signal [MCArt2a–b] > metabolite signal) to not detectable.

In this study, several hydroxylated metabolites of CUMYL-THPINACA and one of ADAMANTYL-THPINACA were prone to in-source dehydration, in most cases attributable to the instability of the hydroxylated 4-methyl-tetrahydropyran-moiety. This most likely resulted in the identification of several artefacts that are discussed in the corresponding chapters referring to the genuine metabolites. In addition, several signals were detected lacking a hydroxylated counterpart, therefore not meeting the above-stated criteria for in-source water loss—they were thus classified as genuine metabolites produced by hydroxylation and desaturation (MC3, MC6, MC12, MC17, MA3, MA8, MA11) or carbonylation (MC13, MC15, MC18, MC20, MC22, MA13, MA10). However, the possibility remains, that the hydroxylated original metabolite was prone to complete in-source water loss, i.e., the original parent ion was no longer detectable. In the context of analytics and the herein presented aims, the focus of this study lies in the identification of suitable biomarkers, which may include highly abundant artefacts resulting from true metabolites. As in-source fragmentation is often seen as an unwanted ESI byproduct, it has also been proposed that in-source-fragment information can improve metabolite identification [44]. However, it must be kept in mind, that the occurrence of in-source-fragmentation processes may also depend on the instrument used, instrument configurations, and ESI conditions.

### 2.3. Metabolic Profiling of CUMYL-THPINACA

**Figure 1:**
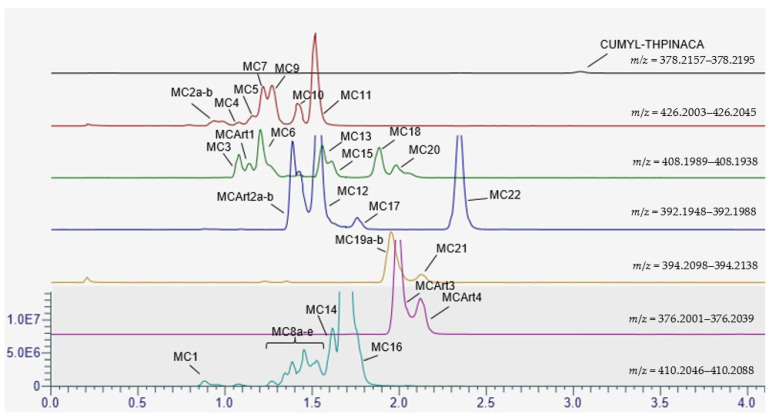
Chromatogram showing the mass traces of the detected metabolites (and artefacts) of CUMYL-THPINACA after 2 h of incubation. The traces are normalized globally, with a maximum at 12% of the base peak (MC16).

**Figure 2:**
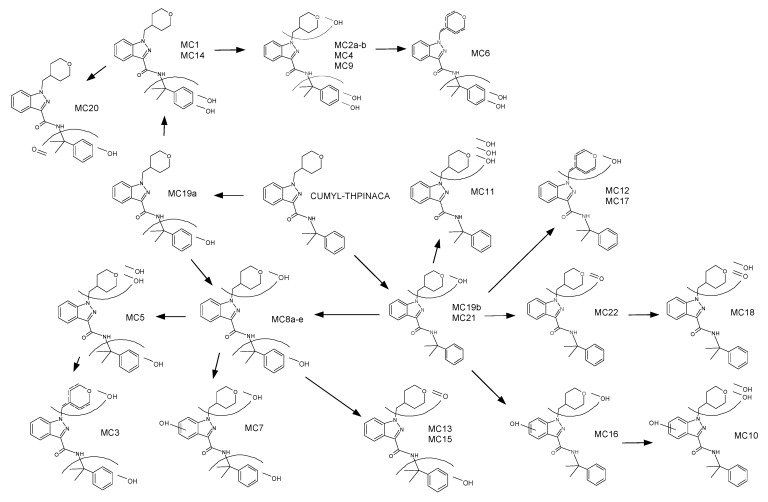
Proposed metabolic pathway for CUMYL-THPINACA.

**Figure 3:**
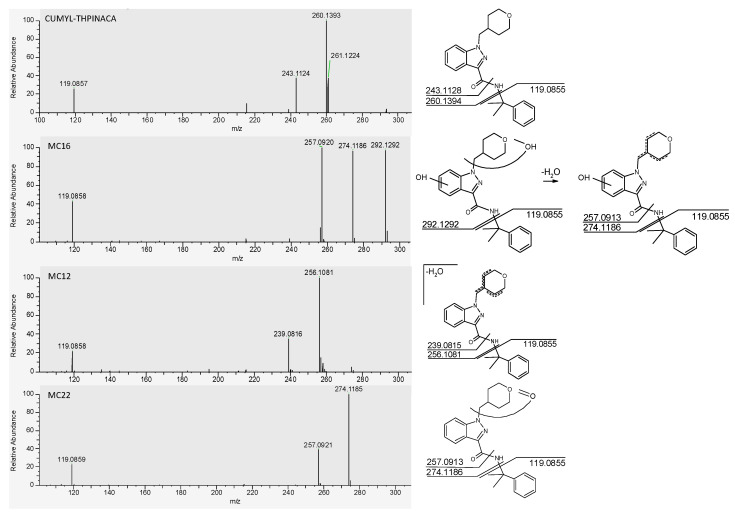
MS^2^ spectra of CUMYL-THPINACA and its three most abundant metabolites (MC12, MC16, and MC22, shown in order of decreasing intensity). The proposed fragments leading to the respective signals are shown on the right.

The fragmentation of CUMYL-THPINACA resulted in three diagnostic fragments at *m*/*z* 119.0855, representing the cumyl-moiety, *m*/*z* 260.1394, referring to the unaltered 1-(tetrahydropyranyl-4-methyl)-indazole-3-carboxamide structure, and *m*/*z* 243.1128, representing the 1-(tetrahydropyranyl-4-methyl)-indazole-3-acylium-ion. A total of three mono-hydroxylated (MC19a–b, MC21), eight di-hydroxylated (MC1, MC8a–e, MC14, MC16), and eight tri-hydroxylated (MC2a–b, MC4, MC5, MC7, MC9, MC10, MC11) metabolites were detected (see Table 1). The di-hydroxylated metabolite MC16, presenting with highest peak areas in the conducted experiments, is suggested as a suitable target in screening procedures. Additional minor metabolites were produced via either hydroxylation with concurrent dehydration, referred to as mono-/di-hydroxylated and desaturated metabolites, or carbonylation. In this context, two mono-hydroxylated and desaturated metabolites (MC12, MC17) and two di-hydroxylated and desaturated metabolites (MC3, MC6) were identified. Finally, carbonylation led to the production of one metabolite (MC22) and mono-hydroxylation in combination with carbonylation resulted in four metabolites (MC13, MC15, MC18, MC20). In-source water loss could not be ruled out for some metabolites; thus, these signals were classified as artefacts (MCArt1, MCArt2a–b, MCArt4, MCArt5). Through conduction of a derivatization experiment, employing iodomethane as the methylating agent, the location of the hydroxyl-groups could be narrowed down to the indazole-core. The main site for biotransformation in regard to number of individual metabolites as well as when considering the most abundant metabolites was the 4-methyl-tetrahydropyran-moiety, while oxidation of the cumyl-moiety was less often observed. There are several other studies investigating the metabolism of SCRAs containing a cumyl-moiety [22,23,26]. These aforementioned studies also concluded that the cumyl-moiety was not the main site of metabolism. A chromatogram showing the mass traces of all metabolites is depicted in Figure 1 and the proposed metabolic pathway of CUMYL-THPINACA is visualized in Figure 2. MS^2^ spectra of CUMYL-THPINACA and the three most abundant metabolites, including proposed fragments, are shown in Figure 3.

#### 2.3.1. Mono-Hydroxylation

MC19a is mono-hydroxylated at the cumyl-moiety, as a fragment at *m*/*z* 135.0804 was detected. For M19b and MC21, mono-hydroxylation at the 1-(tetrahydropyranyl-4-methyl)-indazole-3-carboxamide structure was detected instead (*m*/*z* 276.1343). Due to the observation of in-source dehydration (MCArt3 and MCArt4), and as derivatization did not alter the signals of MC19b and MC21, the location of the hydroxyl groups are suggested to be located at the unsaturated 4-methyl-tetrahydropyran-moiety. MCArt3 and MCArt4 are signals matching the criteria defined in chapter 2.2, and thus are classified as artefacts resulting from in-source dehydration of MC19b and MC21. Fragmentation of MCArt3 and MCArt4 showed desaturation at the 4-methyl-tetrahydropyran-moiety (*m*/*z* 258.1237), which corresponds to the location of mono-hydroxylation of the co-eluting metabolites MC19b and MC21.

#### 2.3.2. Di-Hydroxylation

The most abundant metabolite after 2 h of incubation was the di-hydroxylated metabolite MC16. For MC16, the unaltered cumyl-moiety was detected. As a dehydration reaction was identified during fragmentation, resulting in a fragment at *m*/*z* 274.1186, it was concluded that the second hydroxyl-group is located at the 4-methyl-tetrahydropyran-moiety. The location of the second hydroxyl-group at the indazole-core was verified after derivatization. When fragmenting the proposed product of methylation of MC16 at *m*/*z* 424.2231 (mass shift of 14.0157 Da), a specific fragment corresponding to the 1-(tetrahydropyranyl-4-methyl)-indazole-3-carboxamide-moiety which had been di-hydroxylated and methylated, was detected at *m*/*z* 306.1448.

MC1, MC8a–e, and MC16 are additional metabolites resulting from di-hydroxylation. MC1 is produced via di-hydroxylation at the cumyl-moiety, indicated by a fragment at *m*/*z* 151.0754. Several isomers were detected arising from concurrent mono-hydroxylation at the cumyl-moiety and the 1-(tetrahydropyranyl-4-methyl)-indazole-3-carboxamide-moiety, resulting in the metabolite cluster MC8a–e. As the fragment at *m*/*z* 258.1237 was found throughout (produced via water loss from the 4-methyl-tetrahydropyran-moiety), the hydroxyl-group of the metabolites MC8a–e were concluded to be located at the 4-methyl-tetrahydropyran-moiety. Co-eluted with the metabolite cluster MC8a–e, MCArt2a–b (consisting of two fused peaks), was detected. After fragmentation of MCArt2a–b, the location of one hydroxyl-group was identified at the cumyl-moiety and desaturation was found at the 4-methyl-tetrahydropyran-moiety. Due to the matching type and location of the biotransformation between the original metabolites MC8a–e and MCArt2a–b, the origin of the signal leading to MCArt2a–b was defined as in-source water loss. Finally, MC14 is di-hydroxylated at the di-hydroxylated cumyl-moiety, as the diagnostic fragment at *m*/*z* 151.0754 was identified.

#### 2.3.3. Mono-Hydroxylation and Additional Desaturation and Carbonylation

MC12, MC17, and MC22 are all metabolites sharing the parent ion at *m*/*z* 392.1969. As no co-eluting di-hydroxylated metabolites exhibiting the same patterns were detectable (considering the type and location of biotransformation), in-source water loss as the origin of the corresponding signals was ruled out for these metabolites. MC12 and MC17 presented a fragment at *m*/*z* 256.1081 that resulted from the dehydration of the already desaturated 1-(tetrahydropyranyl-4-methyl)-indazole-3-carboxamide-moiety. Due to the observed desaturation, MC14 and MC19 were classified as mono-hydroxylated and desaturated at the 4-methyl-tetrahydropyran-moiety. The absence of phenolic hydroxyl groups was confirmed via derivatization experiments, as no signal decline of the parent ion was observed. MC22 did not present a dehydration reaction during fragmentation as only the fragments with *m*/*z* 274.1186 (desaturated 1-(tetrahydropyranyl-4-methyl)-indazole-3-carboxamide structure) and *m*/*z* 257.09134 (acylium-ion after cleavage of the C–N bond) were detected. Therefore, M22 was concluded to be carbonylated at the 4-methyl-tetrahydropyran-moiety.

#### 2.3.4. Tri-Hydroxylation

MC2a–b and MC4 are di-hydroxylated at the cumyl-moiety, as verified by detection of the fragment at *m*/*z* 151.0754. MC2a–b and MC4 also present a fragment at *m*/*z* 408.1918 as a result of water loss during fragmentation of the otherwise intact structure. Due to the observed water loss, the location of the one hydroxyl group is situated at the 4-methyl-tetrahydropyran-moiety. MC5 was observed to be mono-hydroxylated at the cumyl-moiety, showing the diagnostic fragment at *m*/*z* 135.0804. The additional fragment at *m*/*z* 256.1081, resulting from two dehydration reactions of the di-hydroxylated 1-(tetrahydropyranyl-4-methyl)-indazole-3-carboxamide structure, verifies the positions of the two other hydroxyl groups at the 4-methyl-tetrahydropyran-moiety. In-source water loss of MC5, leading to the signal of MCArt1, could not be ruled out, due to the proximity of MC5 and the observed signal of MCArt1, which also has one hydroxyl-group at the cumyl-moiety (*m*/*z* 135.0804) but is hydroxylated and additionally desaturated at the tetrahydropyran-moiety. Thus, MCArt1 was defined as a possible artefact.

Mono-hydroxylation at the cumyl-moiety was also observed for MC7. As for MC7, only one dehydration reaction was detected, indicated by the fragment at *m*/*z* 274.1186. Observed fragments for MC7 indicated mono-hydroxylation at the cumyl-moiety, the indazole-core, and at the 4-methyl-tetrahydropyran-moiety. This was also confirmed via the derivatization experiment, as the methylated product of MC7 was detected, presenting a diagnostic fragment at *m*/*z* 306.1448, which represents the di-hydroxylated and methylated 1-(tetrahydropyranyl-4-methyl)-indazole-3-carboxamide-moiety. MC9 is di-hydroxylated at the cumyl-moiety, as shown by the fragment at *m*/*z* 151.0754. Additionally, a fragment at *m*/*z* 258.1237 was detected, which was the dehydration product of the 1-(tetrahydropyranyl-4-methyl)-indazole-3-acylium-ion, thus indicating the location of the third hydroxyl group at the 4-methyl-tetrahydropyran-moiety. MC10 is suggested to be di-hydroxylated at the 4-methyl-tetrahydropyran-moiety, but additionally mono-hydroxylated at the indazole-core. Further, an ion corresponding to the product of tri-hydroxylation and methylation of MC10 at *m*/*z* 440.2180 was detected after derivatization. Fragmentation of this methylated metabolite produced a diagnostic ion at *m*/*z* 322.1397, referring to the methylated tri-hydroxylated 1-(tetrahydropyranyl-4-methyl)-indazole-3-acylium-ion, and thus verifying the location of one hydroxyl group at the unsaturated indazole-region. MC11 is tri-hydroxylated at the 1-(tetrahydropyranyl-4-methyl)-indazole-3-carboxamide structure, as the fragment standing for the tri-hydroxylated 1-(tetrahydropyranyl-4-methyl)-indazole-3-carboxamide-moiety (*m*/*z* 308.1241) was detected. Additionally, this moiety produced further fragments, after one (*m*/*z* 290.1135), two (*m*/*z* 272.1030), and three dehydrations (*m*/*z* 254.0924). Derivatization did not result in a decline of the MC11 signal, thus confirming the location of all three hydroxyl-groups at the unsaturated 4-methyl-tetrahydropyran-moiety.

#### 2.3.5. Mono-Hydroxylation and Additional Desaturation and Carbonylation

MC3 is most likely formed via metabolic tri-hydroxylation (MC5) and concurrent dehydration, resulting in a di-hydroxylated and desaturated molecule. MC3 presented a fragment at *m*/*z* 135.0804, which represents the mono-hydroxylated cumyl-moiety. The desaturation reaction and the second hydroxyl group are located at the 4-methyl-tetrahydropyran-moiety, as the fragment at *m*/*z* 256.1081 was detected—resulting from additional dehydration of this moiety. For MC6, the location of both hydroxyl-groups was found to be at the cumyl-moiety, as a fragment at *m*/*z* 151.0754 was detected. An additionally observed fragment at *m*/*z* 258.1237 was attributed to the desaturated 1-(tetrahydropyranyl-4-methyl)-indazole-3-acylium-ion.

MC13 and MC15 are classified as mono-hydroxylated and carbonylated metabolites. With the diagnostic ion at *m*/*z* 135.0804, both are identified as to be mono-hydroxylated at the cumyl-moiety. Due to the lack of the dehydration reaction during occurring fragmentation, it is suggested that MC13 and MC15 are therefore carbonylated at the 4-methyl-tetrahydropyran-moiety. Mono-hydroxylation in combination with carbonylation was also observed for MC18. Due to the presence of the fragment at *m*/*z* 119.0855, and as MC18 was not methylated during derivatization, proving the absence of phenolic hydroxyl groups, it is assumed that hydroxylation and carbonylation must be located at the 4-methyl-tetrahydropyran-moiety. The fragment at *m*/*z* 290.1135 represents the mono-hydroxylated and carbonylated 1-(tetrahydropyranyl-4-methyl)-indazole-3-carboxamide structure. The fragment at *m*/*z* 272.1030 is produced by additional dehydration and a fragment at *m*/*z* 273.087 results from nitrogen cleavage. MC20 presented two diagnostic ions at *m*/*z* 260.1394 and *m*/*z* 243.1128, representing the unaltered 1-(tetrahydropyranyl-4-methyl)-indazole-3-carboxamide-moiety. The fragment at *m*/*z* 149.0597 represents the cumyl-moiety that has been mono-hydroxylated and carbonylated. With the methylated product of MC21, the location of the hydroxyl was assigned at the phenyl-ring of the cumyl-moiety. In-source water loss, resulting in an additional signal associated with MC3, MC6, MC13, MC17, MC18, and MC20, was not further considered, due to the lack of corresponding tri-hydroxylated metabolites in the respective elution windows. The observed later elution of metabolites MC18 and MC20 was in concordance with the suggested carbonylation, as this biotransformation would result in less polar metabolites compared to the product of di-hydroxylation and desaturation.

#### 2.3.6. Identification of the Primarily Involved CYP Isoenzymes

The results obtained showed that CYP3A4 and CYP3A5 are primarily involved in the metabolism of CUMYL-THPINACA, followed by CYP2D6, CYP2C8, and, to a much lesser extent, by CYP2C19. The involvement of CYP2C9, CYP1A2, and CYP2B6 was also observed. Signals at the retention times of MCArt3 and MCArt4, presenting an area ratio (peak area/internal standard (ISTD) area) of <0.1, were detected in the negative control as well as in the incubation mixture of CYP2E1 and CYP2A6. As these were the only signals detected for CYP2E1 and CYP2A6, it suggests that CYP2E1 and CYP2A6 did not show any metabolic activity for CUMYL-THPINACA. Two metabolites (MC4 and MC5), which were observed after incubation using pHLM, could not be detected after incubation with rCYP (tested with different incubation times), most likely due to these metabolites being produced via a more complex pathway; the involvement of a combination of different CYP; or at concentration levels below the limit of detection. These results are summarized in Table 2.

CYP3A4 is an important CYP isoform with regard to abundancy in the human liver as well as the majority of drugs being known to be substrates of CYP3A4. Overall, due to the primary involvement of CYP3A4, drug−drug interactions may be observable in combination with strong CYP3A4 inhibitors (e.g., azole-antifungals) [45]. Nevertheless, due to the involvement of further CYP isoforms (e.g., CYP2D6, CYP2C8, and CYP2C19), this risk is most likely reduced.

### 2.4. Metabolite Identification for ADAMANTYL-THPINACA

ADAMANTYL-THPINACA was prone to hydroxylation at the adamantyl-moiety and, to a lesser extent, at the 1-(tetrahydropyranyl-4-methyl)-indazole-3-carboxamide-moiety. Extensive hydroxylation at the adamantyl-moiety has also been observed for other adamantyl-bearing SCRAs [31,46,47]. Overall, one mono-hydroxylated (M12), three di-hydroxylated (MA5, MA7, MA9), and four tri-hydroxylated (MA1, MA2, MA4, MA6) were detected. During analysis, two artefacts were detected (MAArt1 and MAArt2), resulting from in-source water loss of di- and a tri-hydroxylated metabolites. In-source water loss was ruled out for three additional metabolites produced via hydroxylation with concurrent desaturation (MA3, MA8, MA11) and mono-hydroxylation in combination with carbonylation (MA10 and MA13). In this study, a total of 13 metabolites were detected for ADAMANTYL-THPINACA (see Table 3). Kadomura et al. reported nine metabolites resulting from phase I metabolism and two glucuronidated metabolites resulting from phase II metabolism [31]. Regarding the previously reported phase I metabolites produced via mono-, di-, and tri-hydroxylation, the results of our study are in good agreement with theirs, with the exception of one additional tri-hydroxylated metabolite that was not detected in our study. This was most probably due to insufficient chromatographic resolution. However, Kadomura et al. did not present the herein observed metabolites produced from hydroxylation in combination with desaturation (MA3, MA8, MA11) and the two mono-hydroxylated and carbonylated metabolites (MA10 and MA13). In the presented study, MA3 and MA8 were ranked as the third and fourth most abundant metabolites, while the rest could be considered as minor metabolites, and thus more likely of limited importance as biomarkers in the in vivo setting. In contrast to Kadomura et al. we did not study the phase II metabolism.

**Figure 4:**
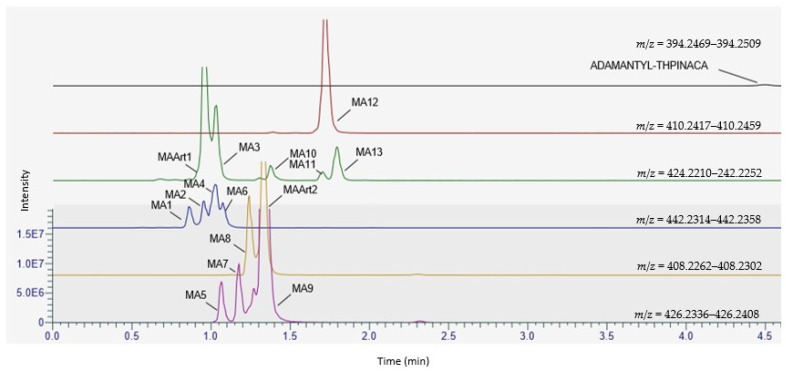
Chromatogram showing the mass traces of the detected metabolites (and artefacts) of ADAMANTYL-THPINACA after 2 h of incubation. The traces are normalized globally, with a maximum at 12% of the base peak (MA9).

**Figure 5:**
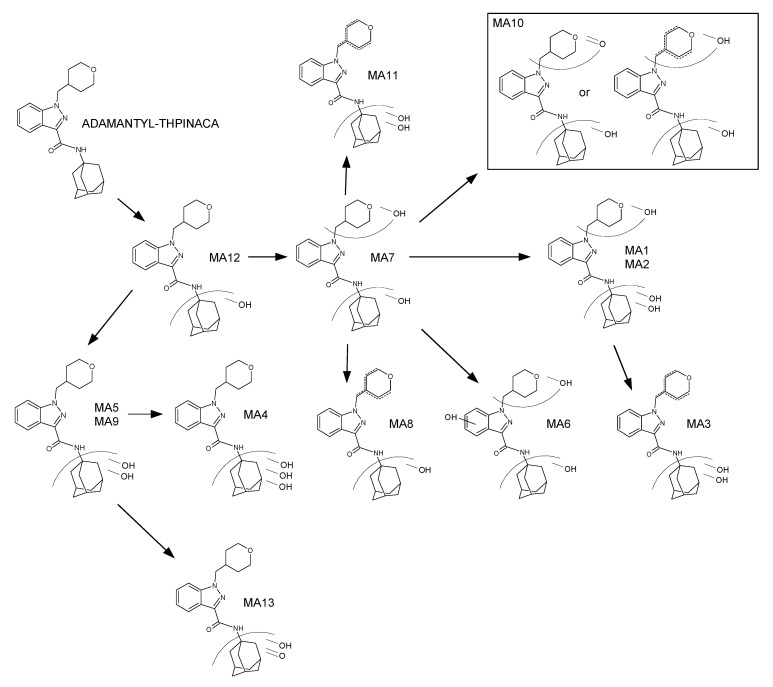
Proposed metabolic pathway of ADAMANTYL-THPINACA.

**Figure 6:**
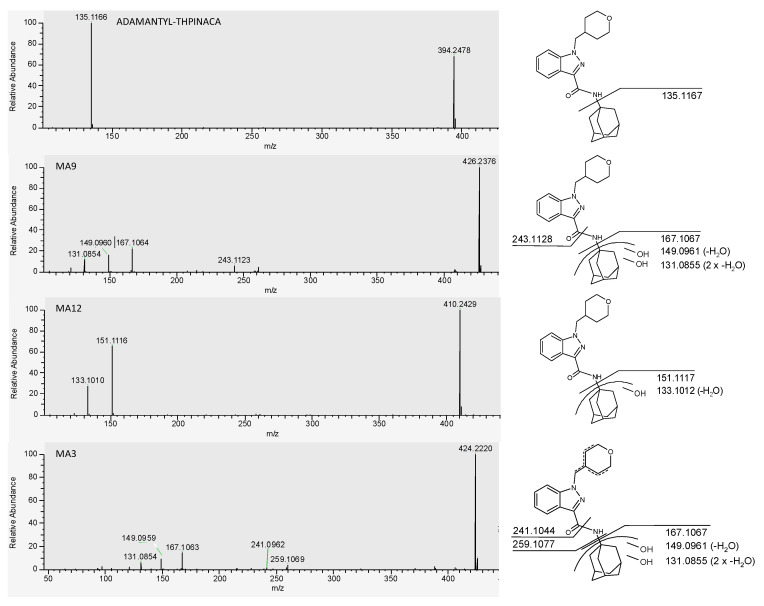
MS2 spectra of ADAMANTYL-THPINACA and its three most abundant metabolites. The proposed fragments leading to the respective signals are shown on the right.

A chromatogram showing the mass traces of all the above-mentioned metabolites and signals in this study is given in Figure 4**.** Due to the high abundancy of the di-hydroxylated metabolite MA9, this metabolite is suggested as a suitable biomarker for urine screenings. Nevertheless, due to limitations of in vitro models, verification in vivo by ana-lysis of positive human urine samples is needed. The proposed metabolic pathway is presented in Figure 5. Fragmentation of the parent compound ADAMANTYL-THPINACA resulted in only one fragment at *m*/*z* 135.1168. Variation of the collision energies did not result in more diagnostic ions for the parent compound (data not shown). Additional diagnostic fragments were detected for the metabolites of ADAMANTYL-THPINACA. The respective MS^2^ spectra of ADAMANTYL-THPINACA, incorporating the three most abundant metabolites with their suggested fragments, are shown in Figure 6.

#### 2.4.1. Mono-Hydroxylation

MA12 is produced via mono-hydroxylation at the adamantyl-moiety, as shown by the diagnostic fragment at *m*/*z* 151.1117.

#### 2.4.2. Di-Hydroxylation

For MA7, the observed fragment of *m*/*z* 151.1117 indicated mono-hydroxylation at the adamantyl-moiety. Therefore, the second hydroxyl group is located at the rest of the molecule. Because derivatization did not change the signal intensity of the MA7 metabolites, this suggests that the second hydroxyl-group is located at the 4-methyl-tetrahydropyran-moiety. The di-hydroxylated metabolite MA9 was the most abundant metabolite. Due to the fragment observed at *m*/*z* 167.1067, the location of both hydroxyl groups was assigned to the adamantyl-moiety. Additional fragments consisted of two dehydration reactions of the di-hydroxylated adamantyl moiety (*m*/*z* 149.0961 and *m*/*z* 131.0855) and the unaltered 1-(tetrahydropyranyl-4-methyl)-indazole-3-acylium-ion (*m*/*z* 243.1128). Two additional, but less abundant, di-hydroxylated metabolites were detected, of which MA5 showed a similar fragmentation pattern to MA9, thus being di-hydroxylated at the adamantyl-moiety. As MAArt2, presenting fragments at *m*/*z* 149.0961 and *m*/*z* 131.0855 indicating dehydration reactions at the hydroxylated adamantyl-moiety, co-eluted with the metabolite MA9, MAArt2 was classified as an in-source artefact produced by dehydration of MA9.

#### 2.4.3. Mono-Hydroxylation and Additional Desaturation

The metabolite MA8 is produced via mono-hydroxylation at the adamantyl-moiety, indicated by fragment *m*/*z* 151.1117. The observed desaturation was assigned to the rest of the molecule (4-methyl-tetrahydropyran-moiety), even though the corresponding fragment was not detected due to neutral loss. As MA8 did not co-elute with a di-hydroxylated metabolite, which is mono-hydroxylated at the adamantyl-moiety as well as at the 4-methyl-tetrahydropyran-moiety, this signal was classified as a genuine metabolite.

#### 2.4.4. Tri-Hydroxylation

The two early-eluting metabolites, MA1 and MA2, were identified to be di-hydroxylated at the adamantyl-moiety and mono-hydroxylated at the 1-(tetrahydropyranyl-4-methyl)-indazole-3-carboxamide structure. For these two metabolites, the observed fragment at *m*/*z* 167.2066 represents the di-hydroxylated adamantyl-moiety and the fragment at *m*/*z* 259.1077 denotes the mono-hydroxylated 1-(tetrahydropyranyl-4-methyl)-indazole-3-acylium-ion. As derivatization did not result in methylation of MA1 and MA2, it was concluded that both metabolites are produced via hydroxylation at the 4-methyl-tetrahydropyran-moiety. MAArt1 was detected via the parent ion at *m*/*z* 424.2231 and is denoted as an in-source dehydration artefact. MAArt1 was identified to be di-hydroxylated at the adamantyl-moiety (*m*/*z* 167.1067) and desaturated at the 4-methyl-tetrahydropyran-moiety (*m*/*z* 259.1077). Due to the presence of the coeluting tri-hydroxylated metabolite MA2, showing the same alterations, a potential contribution from MAArt1 to the observed MA2 signal could not be ruled out. MA4 presented MS^2^ spectra with two fragments at *m*/*z* 260.1393 and *m*/*z* 243.1128, both indicating an unaltered 1-(tetrahydropyranyl-4-methyl)-indazole-3-carboxamide moiety. It was consequently concluded that the adamantyl-moiety was hydroxylated three times, despite the fragment representing this moiety not being detected, due to neutral loss. The latest eluting tri-hydroxylated metabolite MA6 is produced via mono-hydroxylation at the adamantyl-moiety, shown by the diagnostic fragment at *m*/*z* 151.1117, and di-hydroxylation of the remaining molecule. One observed fragment of MA6 at *m*/*z* 274.1184 is produced via dehydration of the 1-(tetrahydropyranyl-4-methyl)-indazole-3-carboxamide-moiety. Therefore, one hydroxyl group must be located at the 4-methyl-tetrahydropyran-moiety. As no second dehydration reaction of this moiety was detected, the third hydroxy group was proposed to be located at the indazole-core. The location of the hydroxyl group at the indazole-moiety was verified via derivatization, as the corresponding methylated metabolite MA6 was detected at *m*/*z* 456.2493. Additionally, fragmentation of this product resulted in a fragment with *m*/*z* 288.1343, indicative of the methylated and desaturated 1-(tetrahydropyranyl-4-methyl)-indazole-3-carboxamide-moiety.

#### 2.4.5. Di-Hydroxylation and Additional Desaturation, Mono-Hydroxylation and Additional Carbonylation

Fragmentation of MA3 with [M + H]^+^ 424.2231 (*m*/*z*), resulted in a fragment at *m*/*z* 167.1067, indicating di-hydroxylation at the adamantyl-moiety. Additionally, the desaturated 4-methyl-tetrahydropyran-moiety was identified with the detected *m*/*z* 259.1077, a fragment indicative of the desaturated 1-(tetrahydropyranyl-4-methyl)-indazole-3-carboxylicacid-moiety after amide hydrolysis. Due to the lack of a tri-hydroxylated counterpart, in-source dehydration was not considered for MA3. The metabolite MA10 resulted in a fragment at *m*/*z* 151.1117, representing the mono-hydroxylated adamantyl-moiety. A fragment produced from subsequent water loss at the adamantyl-moiety was also detected at *m*/*z* 133.1012. Due to a lack of further fragments, as a result of neutral loss, it was concluded that further sites of biotransformation are located elsewhere on the molecule. Potential biotransformations resulting in the signal at *m*/*z* 424.2231 include di-hydroxylation and desaturation (likely derived from dehydration of a tri-hydroxylated metabolite, which was not detected) or mono-hydroxylation in combination with carbonylation. As derivatization did not result in a decrease of the MA10-signal, hydroxylation at the indazole-region was ruled out. In conclusion, MA10 was defined as the product of mono-hydroxylation at the adamantyl-region with concurrent mono-hydroxylation and desaturation or carbonylation at the 4-methyl-tetrahydropyran-moiety. Due to the later elution of MA10, when compared to the detected tri-hydroxylated metabolites, in-source dehydration was not considered. MA11 is a further metabolite with a parent ion at *m*/*z* 424.2231, in this case as a result of di-hydroxylation and desaturation, as indicated by the detection of the di-hydroxylated adamantyl-moiety at *m*/*z* 167.1067. As this fragment was observed, the location of desaturation was concluded to be at the 4-methyl-tetrahydropyran-moiety. As no corresponding tri-hydroxylated metabolites were detected within the MA11 elution window, in-source dehydration of this metabolite is unlikely. MA13 is classified as a product of mono-hydroxylation and carbonylation. This was concluded from the presence of *m*/*z* 260.1393 (unaltered 1-(tetrahydropyranyl-4-methyl)-indazole-3-carboxamide structure) and *m*/*z* 165.0910 (mono-hydroxylation and carbonylation of the adamantyl-moiety). An additional fragment (*m*/*z* 119.0855) was detected, assigned to the cleavage of CO and dehydration of the mono-hydroxylated and carbonylated adamantyl-moiety. The longer retention time of this metabolite when compared to hydroxylated and desaturated metabolites is also in accordance with carbonylation, due to the expected lower polarity of a carbonyl group in comparison to a hydroxyl group.

#### 2.4.6. Identification of the Primarily Involved CYP Isoenzymes

As for CUMYL-THPINACA, CYP3A4 and CYP3A5 were found to mainly contribute to the metabolism of ADAMANTYL-THPINACA (Table 4). In contrast to CUMYL-THPINACA, limited metabolic activity of CYP2D6, and CYP2C8 was observed. CYP2C9 and CYP2C19 mediated the production of M12, but no other metabolites, thus leading to the conclusion that these isoforms play a minor role in the metabolism of ADAMANTYL-THPINACA. For CYP2B6, CYP1A2, CYP2E1 und CYP2A6, no metabolic activity could be observed.

Experiments revealed CYP3A4 also to be the major metabolizing CYP for ADAMANTYL-THPINACA. In comparison to CUMYL-THPINACA, far fewer CYP isoforms were involved in ADAMANTYL-THPINACA metabolism. The intake of strong CYP3A4 inhibitors together with ADAMANTYL-THPINACA is more likely to pose a higher risk of metabolic drug−drug interactions than CYP3A4 in combination with CUMYL-THPINACA.

## 3. Materials and Methods

### 3.1. Chemicals and Reagents

LC-MS grade acetonitrile (ACN), methanol (MeOH), and water, and HPLC grade acetone and 2-isopropanol (IPA) were obtained from Macherey-Nagel AG (Oensingen, Switzerland). Ammonium formate (>99.0%), ethyl acetate (EtOAc, HPLC grade), iodomethane (stabilized with silver), and formic acid (98–100%) were purchased from Merck (Zug, Switzerland). Potassium carbonate (Ph. Eur.) was purchased from Carl Roth AG (Arlesheim, Switzerland). Certified reference standards of UR-144, UR-144 N-(5-hydroxypentyl) metabolite, UR-144 N-pentanoic acid metabolite, and d,l-11-Hydroxy-THC-D_3_ were purchased from Lipomed AG (Arlesheim, Switzerland). CUMYL-THPINACA (N-(1-methyl-1-phenylethyl)-1-(tetrahydropyran-4-ylmethyl)-1H-indazole-3-carboxamide; purity >97%) and ADAMANTYL-THPINACA (N-(1-adamantyl)1-(tetrahydropyran-4-ylmethyl)-1H-indazole-3-carboxamide; purity >93%) were kindly provided by the Zurich Forensic Science Institute (Switzerland) in solid form (as certified reference substances were not available at the time of this work). Stock solutions were prepared at 1 mg/mL in MeOH for both compounds and stored at −20 °C until use. Pooled human liver microsomes (donor pool > 20, 20 mg/mL protein content in 250 mM sucrose, specified total P450 enzyme content of 360 pmol/mg protein) and Gentest NADPH regenerating system solutions: A (containing 26 mM β-nicotinamide adenine dinucleotide phosphate [NADP^+^], 66m MD-glucose-6-phosphate [Glc-6-P], 66mMmagnesium chloride [MgCl_2_] in water), and B (containing 40 U/mL Glc-6-P dehydrogenase [Glc-6-P-DH; EC 1.1.1.49] in sodium citrate) were purchased from Corning (Amsterdam, The Netherlands). Human CYP3A4 (100 pmol/mg protein), CYP2C9 (100 pmol/mg protein), CYP2E1 (100 pmol/mg protein), CYP3A5 (100 pmol/mg protein), CYP2C8 (100 pmol/mg protein), CYP2B6 (100 pmol/mg protein), CYP2A6 (with purified human cytochrome b5, 100 pmol/mg protein), CYP1A2 (770 pmol/mg protein), CYP2D6 (142 pmol/mg protein), CYP2C19 (202 pmol/mg protein), CYP3A5, and CYP2B6 EasyCYP Bactosomes co-expressed with human CYP-reductase in Escherichia coli, and membrane protein isolated from Escherichia coli host strain (EasyCYP control, 10 mg/mL protein), were ordered from tebu-bio (Offenbach, Germany).

### 3.2. Microsomal Incubation with pHLM

CUMYL-THPINACA, ADAMANTYL-THPINACA were incubated in duplicate at final concentrations of 10 µM in a total reaction volume of 1000 µL. Following the vendors instructions (Corning), the incubation mixture consisted of 100 mM potassium phosphate buffer (pH 7), 50 µL NADPH Regenerating System Solution A, and 10 µL NADPH Regenerating System Solution B. The percentage of organic solvent (MeOH) was limited to 0.4% in the incubation mixture, thus no inhibition due to organic solvents was to be expected (limit for MeOH defined by Corning: 1%). The reaction was started by the addition of 0.5 mg liver microsomes per assay to the reaction mixture that was then tempered to 37 °C. Negative controls were prepared by replacing the pHLMs with an equivalent volume of water and by incubating enzymes without the addition of SCRAs, while parallel incubation of the SCRA UR-144 served as a positive control for the functionality of the incubation. The samples were then incubated at 37 °C in an Eppendorf ThermoStat C heating block. In a preliminary experiment (data not shown) samples were drawn after 0.5 h, 1 h, 1.5 h and 2 h after incubation with pHLM. For the presented data, the samples were incubated for 2 h, as this incubation time gave the best outcome with respect to number and concentration of metabolites. The reaction was terminated by the addition of an equal volume of ice-cold ACN to 400 µL of drawn sample. The samples were then centrifuged at room temperature for five minutes at 13,400 rpm (approximately 9000× *g*) using an Eppendorf MiniSpin centrifuge (Eppendorf, Schönenbuch, Switzerland). The supernatant was stored in glass vials at −20 °C until sample cleanup.

### 3.3. Microsomal Incubation with rCYP

Substrate solutions (final concentration 10 µM) were incubated in 100 mM potassium phosphate buffer (pH 7), containing a final reaction volume of 500 µL, containing 38 µL NADPH regenerating solution A and 13 µL NADPH Regenerating Solution B. The reaction was started by the addition 25 µL of the CYP-solutions (or negative control EasyCYP), resulting in 0.5 mg protein per assay. Following the vendors instructions (tebu-bio), the reaction was quenched—after incubating for 20 min at 37 °C—by addition of 400 µL ice-cold ACN to 400 µL of drawn sample. Subsequently, the samples were centrifuged at 13,400 rpm (approximately 9000× *g*) for five minutes. The supernatant was stored in glass vials at −20 °C until further processing.

### 3.4. Sample Preparation

For sample clean-up, a protocol was adapted from one developed for the analysis of metabolites of SCRAs in urine, published by Gaunitz et al. [35]. In brief, 600 µL of the supernatants of the precipitated samples were diluted 1:1 with 100 mM ammonium formate buffer (pH 4). At this point the internal standard (ISTD) d,l-11-Hydroxy-THC-D_3_ was added, resulting in a concentration of 100 ng/mL (final concentration at time of analysis, with presumed 100% recovery, 300 ng/mL). Strata phenyl SPE cartridges obtained from Phenomenex (Basel, Switzerland) were conditioned with 2 mL MeOH, 2 mL water, and 2 mL ammonium formate buffer (pH 4), prior to being loaded with the diluted samples. After loading the samples, the cartridges were washed with 2 mL of 5:95 MeOH:water (*v*/*v*) and dried for 15 min. Elution of the analytes was achieved with twice 2 mL of 85:15 EtOAc:IPA (*v*/*v*). Extracts were collected in glass tubes and the solvent was evaporated until dryness at 40 °C under a gentle stream of nitrogen. Finally, the dried residues were resolved in 200 µL of 1:1 ACN:water (*v*/*v*), centrifuged (2465× *g*, 15 min) and transferred to HPLC-vials, thus resulting in a concentration by a factor of 3.

### 3.5. Derivatization Using Iodomethane

SPE extracts obtained from pHLM incubation experiments were solved in 200 µL acetone and then transferred into glass-vials, which were prefilled with a spatula tip (approximately 500 mg) of potassium carbonate. At this point 100 µL iodomethane was added, the vials were closed, and the mixtures were incubated for 1 h at 60 °C. The samples were transferred into a new vial using a glass Pasteur pipet omitting the insoluble potassium carbonate. The samples were evaporated to dryness under a gentle nitrogen stream at 60 °C, and reconstituted in 200 µL 1:1 ACN:water. A negative control was conducted for both SCRAs, where the addition of iodomethane was omitted while the rest of the experiment was kept as above.

### 3.6. Analysis

Chromatographic separation of the metabolites was achieved using a Dionex UltiMate 3000 ultra UHPLC system equipped with a Hypersil Gold (50 × 2.1 mm 1.9 µM) analytical column, thermostatted at 40° C using a MutliSLEEVE column heater, all obtained from Thermo Fisher Scientific (Reinach, Switzerland). Mobile phase A consisted of water with 0.1% (*v*/*v*) formic acid and mobile phase B of ACN with 0.1% (*v*/*v*) formic acid. After injection of 5 µL of the prepared sample the gradient commenced at 20% mobile phase B, which then increased to 40% within 0.9 min and to 71% within the following 6 min, after which the mobile phase B was increased to 100% during a time interval of 0.25 min and held for 1 min. The system was then returned to the initial settings and held for 1.25 min, prior to the injection of the next sample. The mobile phase flow was 0.6 mL/min throughout. The mobile phase flow during the first 0.1 min and after 7 min was directed to the waste and not to the mass spectrometer by means of a bypass valve connected after the column.

Subsequent analysis was undertaken with a Thermo Scientific Q Exactive HF Hybrid Quadrupole-Orbitrap mass spectrometer equipped with a heated electrospray ionization (HESI-II) source, obtained from Thermo Fisher Scientific (Reinach, Switzerland), operated with a sheath gas flow rate of 50 arbitrary units (AU) and an auxiliary gas flow rate of 5 AU. The capillary temperature and auxiliary gas heater temperature were 200 °C and 350 °C, respectively, and the spray voltage was set to 3.5 kV. Parent ions of metabolites were screened using a full MS acquisition in positive ion mode and at a resolution of 120,000 full width at half-maximum (FWHM) at *m*/*z* 200, within a scan range from *m*/*z* 150 to *m*/*z* 1000. Metabolite identification was conducted by manual investigation of the raw data in FreeStyle (version 1.7, SP1, Thermo Fisher Scientific, Reinach, Switzerland), assisted by the Compound Discoverer (version 3.1, Thermo Fisher Scientific, Reinach, Switzerland) software, by running an expected workflow (Forensics Expected w FiSh scoring), that enables the screening for software predicted products generated by biotransformation of a predefined compound. The software thus calculates the expected masses of common phase I metabolites and searches for corresponding signals in the data. The program additionally identifies background signals by comparison of blank samples and negative control samples, which are then filtered out and, therefore, not considered.

In a subsequent analysis, the software-proposed metabolites were transferred into an inclusion list for a full MS—data-dependent MS^2^ (dd-MS^2^) analysis. The resolution for this measurement was set to 60,000 FWHM for the full MS analysis and 15,000 FWHM for the dd-MS^2^ analysis. Normalized stepped collision energies of 10, 17.5, and 35 (normalized to *m*/*z* 500 [z = 1]) were applied. In order to ensure fragmentation of low abundance and close eluting isobaric compounds, the minimum automated33 gain control (AGC) target to trigger an MS^2^ measurement was set to zero and dynamic exclusion was set to one second. The generated MS^2^ spectra were investigated with the aid of Compound Discoverer, which enables comparison of the obtained MS^2^ spectra to the theoretical in silico generated MS^2^-spectra [38].

The criterion for metabolite identification was a mass accuracy <5 ppm for the proposed parent ions and diagnostic fragments along with the plausibility of observed fragments and observed retention times of metabolites in relation to each other. The biotransformations were identified by mass shifts of the detected fragments, indicative of hydroxylation (+15.9994 Da per hydroxylation), desaturation (−2.01565 Da), carbonylation (+13.9838 Da), dehydration (−18.0153 Da), and combinations thereof. The position of hydroxylation was narrowed down by a derivatization experiment employing iodomethane, which selectively methylates aromatic hydroxyl-groups (i.e., cumyl- and indazole-moiety).

## 4. Conclusions

Incubation with pHLM yielded 28 metabolites for CUMYL-THPINACA and 13 metabolites for ADAMANTYL-THPINACA. The observed extensive metabolism of the studied SCRAs again highlight, as previously observed for many other SCRAs, the need to include the metabolites in screening procedures—particularly in urine. Both compounds presented a highly abundant di-hydroxylated metabolite, which is recommended as a suitable target for screening procedures. For both compounds, in-source dehydration artefact formation was observed for a few hydroxylated metabolites, supporting the need for in vitro studies prior to moving on to in vivo measurements. However, several metabolites, sharing the same mass as the described dehydration artefacts, were identified. This emphasizes the requirement to thoroughly investigate all signals of potential metabolites in order not to miss potential biomarkers. Furthermore, as some dehydration products presented higher abundancies than the underlying metabolite, their detection may improve investigations of substance use and they should, therefore, be included into screening protocols. However, such recommendations would need to be verified by means of analysis of human urine samples.

The reported protocols, along with the instrumentation and software used, proved to be beneficial for the investigation of SCRA metabolite profiles. The in silico tools were invaluable in speeding up the elucidation of the metabolic profile of the studied SCRAs. Concerning the metabolism, the involvement of mainly CYP3A4 along with CYP3A5, was observed for both compounds. For CUMYL-THPINACA the additional involvement of 2D6, 2C8, and 2C19 was found (all to a lesser extent than for CYP3A4), making CUMYL-THPINACA less susceptible for metabolism-based drug−drug interactions or the effects of CYP-polymorphism. Due to the main involvement of CYP3A4 in the metabolism of ADAMANTYL-THPINACA, metabolic drug−drug interactions in combination with a strong CYP3A4 inhibitor are considered more likely with ADAMANTYL-THPINACA than with CUMYL-THPINACA.

## Figures and Tables

**Table 1:** Summary of all detected metabolites and the two detected artefacts of CUMYL-THPINACA (listed in the order of the observed retention times) with suggested biotransformations, chemical formulas, calculated [M + H]^+^ of the parent ions with associated product ions (*m*/*z*), mass error (ppm), retention times (Rt), areas after 2 h of incubation using pHLM and ranking (highest to lowest abundancy).

ID	Biotransformation	Formula	[M + H]^+^ Productions (*m*/*z*)	Mass Error (ppm)	Rt (min)	Area (*n* = 2)	Rank
MC1	di-hydroxylation at cumyl	C_23_H_28_N_3_O_4_	410.2074	0.4	0.89	3.0 × 10^6^	21
			259.1077				
			151.0754				
MC2a–b	di-hydroxylation at cumyl, mono-hydroxylation at 4-methyl-tetrahydropyran	C_23_H_28_N_3_O_5_	426.2023	0.4	a: 0.90	3.9 × 10^6^	19
			408.1918				
			259.1077		b: 0.99		
			151.0754				
MC3	mono-hydroxylation at cumyl, mono-hydroxylation and desaturation at 4-methyl-tetrahydropyran	C_23_H_28_N_3_O_5_	408.1918	−0.1	1.08	8.00 × 10^6^	14
			256.1081				
			135.0804				
MC4	di-hydroxylation at cumyl, mono-hydroxylation at 4-methyl-tetrahydropyran	C_23_H_28_N_3_O_5_	426.2023	−0.2	1.08	1.1 × 10^6^	22
			408.1918				
			259.1077				
			151.0754				
MCArt1	In-source water loss of MC5	C_23_H_28_N_3_O_5_	408.1918	0.4	1.16	4.5 × 10^6^	-
			256.1081				
			135.0804				
MC5	mono-hydroxylation at cumyl, di-hydroxylation at 4-methyl-tetrahydropyran	C_23_H_28_N_3_O_5_	426.2023	0.8	1.19	3.7 × 10^6^	20
			274.1186				
			256.1081				
			135.0804				
MC6	di-hydroxylation at cumyl, desaturation at 4-methyl-tetrahydropyran	C_23_H_28_N_3_O_5_	408.1918	−0.3	1.20	2.3 × 10^7^	7
			258.1237				
			151.0754				
MC7	mono-hydroxylation at cumyl, mono-hydroxylation at indazole, mono-hydroxylation at 4-methyl-tetrahydropyran	C_23_H_28_N_3_O_5_	426.2023	−0.6	1.23	1.6 × 10^7^	10
			292.1292				
			274.1186				
			257.0913				
			135.0804				
MC8a–e	mono-hydroxylation at cumyl and mono-hydroxylation at 4-methyl-tetrahydropyran	C_23_H_28_N_3_O_4_	410.2074	−1.3	a: 1.28 b: 1.35 c: 1.39 d: 1.46 e: 1.53	4.4 × 10^7^	4
			258.1237				
			241.0972				
			135.0804				
MC9	di-hydroxylation at cumyl, mono-hydroxylation at 4-methyl-tetrahydropyran	C_23_H_28_N_3_O_5_	426.2023	−0.6	1.27	1.9 × 10^7^	9
			258.1237				
			151.0754				
MCArt2a–b	In-source water loss MC8a–e	C_23_H_26_N_3_O_3_	392.1969	−1.7	a: 1.39	5.5 × 10^7^	-
			258.1237				
			241.0972		b: 1.44		
			135.0804				
MC10	di-hydroxylation at 4-methyl-tetrahydropyran, mono-hydroxylation at indazole	C_23_H_28_N_3_O_5_	426.2023	−0.7	1.42	9.1 × 10^6^	13
			308.1241				
			290.1135				
			272.103				
			119.0855				
MC11	tri-hydroxylation at 4-methyl-tetrahydropyran	C_23_H_28_N_3_O_5_	426.2023	−0.5	1.51	3.8 × 10^7^	5
			308.1241				
			290.1135				
			272.103				
			254.0924				
			119.0855				
MC12	mono-hydroxylation and desaturation at 4-methyl-tetrahydropyran	C_23_H_26_N_3_O_3_	392.1969	−1.6	1.54	7.8 × 10^7^	2
			256.1081				
			239.0815				
MC13	mono-hydroxylation at cumyl, carbonylation at 4-methyl-tetrahydropyran	C_23_H_28_N_3_O_5_	408.1918	−1.2	1.56	1.1 × 10^7^	12
			274.1186				
			257.0913				
			135.0804				
MC14	di-hydroxylation at cumyl	C_23_H_28_N_3_O_4_	410.2074	−1.1	1.62	2.3 × 10^7^	8
			260.1394				
			151.0754				
MC15	mono-hydroxylation at cumyl, carbonylation at 4-methyl-tetrahydropyran	C_23_H_28_N_3_O_5_	408.1918	−0.1	1.62	6.3 × 10^6^	16
			274.1186				
			257.0913				
			135.0804				
MC16	mono-hydroxylation at 4-methyl-tetrahydropyran, mono-hydroxylation at indazole	C_23_H_28_N_3_O_4_	410.2074	−1.3	1.69	3.4 × 10^8^	1
			292.1292				
			274.1186				
			257.0913				
			119.0855				
MC17	mono-hydroxylation and desaturation at 4-methyl-tetrahydropyran	C_23_H_26_N_3_O_3_	392.1969	−0.9	1.81	5.6 × 10^6^	17
			256.1081				
			239.0815				
			119.0855				
MC18	mono-hydroxylation and carbonylation at 4-methyl-tetrahydropyran	C_23_H_28_N_3_O_5_	408.1918	−0.2	1.88	1.3 × 10^7^	11
			290.1135				
			273.087				
			272.103				
			119.0855				
MC19a–b	a: mono-hydroxylation at cumyl	C_23_H_28_N_3_O_3_	394.2118	1.4	1.99	3.0 × 10^7^	6
		a:	135.0804				
			260.1394				
			243.1128				
	b: mono-hydroxylation at 4-methyl-tetrahydropyran	b:	276.1343				
			119.0855				
MCArt3	In-source water loss MC19b	C_23_H_25_N_3_O_2_	376.2020	−0.7	2.03	1.5 × 10^7^	-
			258.1237				
			119.0855				
MC20	mono-hydroxylation and carbonylation at cumyl	C_23_H_28_N_3_O_5_	408.1918	−0.1	1.98	7.0 × 10^6^	15
			260.1394				
			243.1128				
			149.1660				
MC21	mono-hydroxylation at 4-methyl-tetrahydropyran	C_23_H_28_N_3_O_3_	394.2118	1.4	2.15	4.4 × 10^6^	18
			258.1237				
			119.0855				
MCArt4	In-source water loss MC21	C_23_H_25_N_3_O_2_	376.2020	−0.5	2.17	4.7 × 10^6^	-
			258.1237				
			119.0855				
MC22	carbonylation at 4-methyl-tetrahydropyran	C_23_H_26_N_3_O_3_	392.1969	−1.3	2.39	5.0 × 10^7^	3
			274.1186				
			257.0913				
			119.0855				
CUMYL-THPINACA		C_23_H_27_N_3_O_2_	378.2176	0.1	3.07	8.9 × 10^5^	-
			260.1394				
			243.1128				
			119.0855				

**Table 2:** Results of the incubation of CUMYL-THPINACA with rCYP. Listed area ratios (absolute peak are (metabolite)/absolute peak area (ISTD)) are classified as follows: (+): <0.1, +: ≥0.1–≤1, ++: >1–≤5, +++: >. Where the negative control contained trace amounts of metabolites in a comparable amount, as in the samples after incubation, the result is marked as: (+) *.

ID	3A4	3A5	2D6	2C8	2C9	2C19	2B6	1A2	2E1	2A6	Negative Control
	*n* = 2	*n* = 2	*n* = 2	*n* = 2	*n* = 2	*n* = 2	*n* = 2	*n* = 2	*n* = 2	*n* = 2	*n* = 2
MC1	(+)	(+)	-	-	-	-	-	-	-	-	-
MC2a–b	+	(+)	-	-	-	-	-	-	-	-	-
MC3	(+)		-	-	-	-	-	-	-	-	-
MC4	-	-	-	-	-	-	-	-	-	-	-
MCArt1	(+)	(+)	-	-	-	-	-	-	-	-	-
MC5	-	-	-	-	-	-	-	-	-	-	-
MC6	(+)	(+)	-	-	-	-	-	-	-	-	-
MC7	(+)	(+)	-	-	-	-	-	-	-	-	-
MC8a–e	+	+	(+)	(+)	(+)	(+)	-	-	-	-	-
MC9	(+)				-		-	-	-	-	-
MCArt2a–b	+	+	(+)	(+)	-	(+)	-	-	-	-	-
MC10	+	+	-	-	-	-	-	-	-	-	-
MC11	+	(+)	-	-	-	-	-	-	-	-	-
MC12	++	+	-	-	-	-	-	-	-	-	-
MC13	(+)	-	-	-	-	-	-	-	-	-	-
MC14	+	+	(+)	(+)	-	(+)	-	-	-	-	-
MC15	(+)	-	-	-	-		-	-	-	-	-
MC16	++	++	+	(+)	-	(+)	-	-	-	-	-
MC17	+	(+)	-	-	-	-	-	-	-	-	-
MC18	+	(+)	-	-	-	-	-	-	-	-	-
MC19a–b	+++	++	++	+	(+)	+	(+)	(+)	-	-	-
MCArt3	+++	+++	+++	++	+	++	+	+	(+) *	(+) *	(+) *
MC20	+	(+)	-	-	-	-	-	-	-	-	-
MC21	+++	+++	++	++	+	++	(+)	(+)	-	-	-
MCArt4	+++	+++	+++	+++	+	+++	(+) *	(+) *	(+) *	(+) *	(+) *
MC22	++	+	(+)	(+)	-	(+)	-	-	-	-	-

**Table 3:** Summary of all detected metabolites, and observed artefacts thereof, of ADAMANTY-THPINACA (listed in the order of the observed retention times). Shown are the suggested biotransformations, chemical formulas, calculated [M + H] of the parent ions and the corresponding product ions, as well as retention times, area after 2 h of incubation, and rank.

ID	Biotransformation	Formula	[M + H]^+^ Product ions (*m*/*z*)	Mass error (ppm)	Rt (min)	Area (*n* = 2)	Rank
MA1	di-hydroxylation at adamantyl, mono-hydroxylation at 4-methyl-tetrahydropyran	C_24_H_31_N_3_O_5_	442.2336	1.4	0.87	9.2 × 10^6^	11
			424.2221				
			259.1077				
			167.1067				
			149.0961				
			131.0855				
MA2	di-hydroxylation at adamantyl, mono-hydroxylation at 4-methyl-tetrahydropyran	C_24_H_31_N_3_O_5_	442.2336	−0.7	0.97	1.1 × 10^7^	10
			424.2221				
			259.1077				
			167.1067				
			149.0961				
			131.0855				
MAArt1	in-source water loss of MA2	C_24_H_29_N_3_O_4_	424.2231	−0.7	0.98	7.5 × 10^7^	
			259.1077				
			241.1044				
			167.1067				
			149.0961				
			131.0855				
MA3	di-hydroxylation at adamantyl, desaturation at 4-methyl-tetrahydropyran	C_24_H_29_N_3_O_4_	424.2231	−0.5	1.03	3.8 × 10^7^	3
			259.1077				
			241.1044				
			167.1067				
			149.0961				
			131.0855				
MA4	tri-hydroxylation at adamantyl	C_24_H_31_N_3_O_5_	442. 2336	−0.5			
			424.2231				
			260.1393				
			243.1128				
MA5	di-hydroxylation at adamantyl	C_24_H_31_N_3_O_3_	426.2387	1.2	1.09	1.7 × 10^7^	8
			260.1394				
			243.1128				
			167.1067				
			149.0961				
			131.0855				
MA6	mono-hydroxylation at adamantyl, mono-hydroxylation at indazole, mono-hydroxylation at 4-methyl-tetrahydropyran	C_24_H_31_N_3_O_5_	442.2336	0	1.1	1.3 × 10^7^	9
			424.2221				
			406.2114				
			151.1117				
			133.1012				
			274.1186				
			257.0921				
MA7	mono-hydroxylation at adamantyl, mono-hydroxylation at 4-methyl-tetrahydropyran	C_24_H_31_N_3_O_3_	426.2387	0.5	1.2	2.4 × 10^7^	6
			151.1117				
			133.1012				
MA8	Mono-hydroxylation at adamantyl, desaturation at 4-methyl-tetrahydropyran	C_24_H_29_N_3_O_3_	408.2282	−0.2	1.27	3.2 × 10^7^	4
			151.1117				
			133.10118				
MA9	di-hydroxylation at adamantyl	C_24_H_31_N_3_O_3_	426.2387	−1.2	1.36	4.2 × 10^8^	1
			243.1128				
			167.1067				
			149.0961				
			131.0855				
MAArt2	in-source water loss of MA9	C_24_H_29_N_3_O_3_	408.2282	−0.7	1.36	7.9 × 10^7^	
			260.1394				
			243.1128				
			149.0961				
			131.0855				
MA10	Mono-hydroxylation at adamantyl, and carbonylation or mono-hydroxylation and desaturation at 4-methyl-tetrahydropyran	C_24_H_29_N_3_O_4_	424.2231	0	1.39	6.9 × 10^6^	12
			151.1117				
			133.1012				
MA11	Di-hydroxylation at adamantyl, desaturation at 4-methyl-tetrahydropyran	C_24_H_29_N_3_O_4_	424.2231	−0.9	1.73	4.1 × 10^6^	13
			259.1077				
			167.1067				
			149.0961				
			131.0855				
MA12	mono-hydroxylation at adamantyl	C_24_H_31_N_3_O_3_	410.2438	−0.5	1.75	7.6 × 10^7^	2
			151.1117				
			133.1012				
MA13	carbonylation and mono-hydroxylation at adamantyl	C_24_H_29_N_3_O_4_	424.2231	0.2	1.8	1.8 × 10^7^	7
			406.2125				
			260.1394				
			243.1128				
			165.091				
MA1			119.0855				
ADAMANTYL-THPINACA		C_24_H_31_N_3_O_3_	394.2489	0.6	4.56	1.0 × 10^6^	-
			135.1167				

**Table 4:** Summary of the incubation results of ADAMANTYL-THPINACA with rCYP. Area ratios (absolute peak are (metabolite)/absolute peak area (ISTD)) are expressed as follows: (+): <0.1, +: ≥0.1–≤1, ++: >1–≤5, +++: >5. Where the negative control contained trace amounts of metabolites in a comparable amount, as in the samples after incubation, the result is marked as: (+) *.

ID	3A4	3A5	2D6	2C8	2C9	2C19	2B6	1A2	2E1	2A6	Negative Control
	*n* = 2	*n* = 2	*n* = 2	*n* = 2	*n* = 2	*n* = 2	*n* = 2	*n* = 2	*n* = 2	*n* = 2	*n* = 2
MA1	+	(+)	-	-	-	-	-	-	-	-	-
MA2	+	(+)	-	-	-	-	-	-	-	-	-
MAArt1	++	(+)	-	-	-	-	-	-	-	-	-
MA3	++	(+)	-	-	-	-	-	-	-	-	-
MA4	+	(+)	-	-	-	-	-	-	-	-	-
MA5	+	(+)	(+)	(+)	-	-	-	-	-	-	-
MA6	(+)	(+)	-	-	-	-	-	-	-	-	-
MA7	+	(+)	(+)	(+)	-	-	-	-	-	-	-
MA8	+	++	+	(+)	-	-	-	-	-	-	-
MA9	+++	++	(+)	(+)	-	-	-	-	-	-	-
MAArt2	++	+	(+)	(+)	-	-	-	-	-	-	-
MA10	(+)	(+)	-	-	-	-	-	-	-	-	-
MA11	(+)	(+)	-	-	-	-	-	-	-	-	-
MA12	+	+++	++	+++	+	+	(+) *	(+) *	(+) *	(+) *	(+) *
MA13	+	(+)	-	-	-	-	-	-	-	-	-

## Data Availability

Data is available in the article.
